# Plasmacytoid dendritic cells are short-lived: reappraising the influence of migration, genetic factors and activation on estimation of lifespan

**DOI:** 10.1038/srep25060

**Published:** 2016-04-26

**Authors:** Yifan Zhan, Kevin V. Chow, Priscilla Soo, Zhen Xu, Jamie L. Brady, Kate E. Lawlor, Seth L. Masters, Meredith O’keeffe, Ken Shortman, Jian-Guo Zhang, Andrew M. Lew

**Affiliations:** 1 The Walter & Eliza Hall Institute of Medical Research, Parkville, 3052, Australia; 2 Department of Medical Biology, University of Melbourne, Parkville, 3010, Australia; 3 Department of Nephrology, Royal Melbourne Hospital, Parkville, 3050, Australia; 4 Monash Biomedicine Discovery Institute, Department of Biochemistry and Molecular Biology, Monash University, Clayton VIC 3168, Australia; 5 Department of Microbiology & Immunology, University of Melbourne, Parkville, 3010, Australia

## Abstract

Plasmacytoid dendritic cells (pDCs) play an important role in immunity to certain pathogens and immunopathology in some autoimmune diseases. They are thought to have a longer lifespan than conventional DCs (cDCs), largely based on a slower rate of BrdU labeling by splenic pDCs. Here we demonstrated that pDC expansion and therefore BrdU labeling by pDCs occurs in bone marrow (BM). The rate of labeling was similar between BM pDCs and spleen cDCs. Therefore, slower BrdU labeling of spleen pDCs likely reflects the “migration time” (∼2 days) for BrdU labeled pDCs to traffic to the spleen, not necessarily reflecting longer life span. Tracking the decay of differentiated DCs showed that splenic pDCs and cDCs decayed at a similar rate. We suggest that spleen pDCs have a shorter *in vivo* lifespan than estimated utilizing some of the previous approaches. Nevertheless, pDC lifespan varies between mouse strains. pDCs from lupus-prone NZB mice survived longer than C57BL/6 pDCs. We also demonstrated that activation either positively or negatively impacted on the survival of pDCs via different cell-death mechanisms. Thus, pDCs are also short-lived. However, the pDC lifespan is regulated by genetic and environmental factors that may have pathological consequence.

Dendritic cells (DCs) consist of many subsets (e.g. plasmacytoid DCs = pDCs, conventional DCs = cDCs) that perform different immunological functions. They act as a “double-edge sword” by offering protection against infection but also causing immunopathology/inflammation. Thus DC homeostasis is critical for the balance of immunity and immune tolerance.

DC homeostasis is partly dependent upon the lifespan of individual DC subsets^1^. Estimation of the *in vivo* lifespan of DC subsets has mainly been performed using two approaches. One common approach is to track cell turnover by BrdU labeling: slow labeling indicates longer lifespan while fast labeling indicates shorter lifespan. With this approach, it has been revealed that labeling rates of different DC subsets from different lymphoid organs vary substantially^2^,^3^, suggesting different lifespans for different DC subsets. Relative to the fast BrdU labeling of splenic cDCs, the kinetics of BrdU labelling by splenic pDC is considerably slower, suggesting that pDCs are long-lived cells^4^. This finding has been confirmed independently in two subsequent studies^5^,^6^.

Apart from BrdU labeling, parabiosis experiments have also been performed to assess turnover and lifespan of DC subsets^5^. In this study, the rate of DC turnover was determined by following the disappearance of parabiont-derived DCs after parabiosis was severed. Loss of parabiont-derived pDCs reached background levels within 3 days, even shorter than the loss of parabiont cDCs, therefore suggesting a short lifespan for splenic pDCs^5^. A potential caveat for the model is that trauma induced by surgery may have differential effects on circulating pDCs and lymphoid-resident cDCs. Nevertheless, significant differences in the estimated pDC lifespan between BrdU labeling and parabiosis requires reconciliation in order to establish the true lifespan of peripheral pDCs.

We reason that there are several factors that may influence pDC lifespan and interpretation of the data concerning pDC lifespan. Firstly, final differentiation of pDCs and cDCs occurs at different sites: cDCs in secondary lymphoid organs like the spleen and pDCs in the BM. Thus, unlike cDCs, BrdU labeling of pDCs actually occurs in the BM and slow(er) labeling of BrdU by spleen pDCs might reflect the required migration time from BM to spleen. In support of this, it was found that 5% of splenic cDCs were Ki-67^+^ while <0.5% of pDCs were Ki-67^+^ when expression of the nuclear antigen Ki-67 was used to measure proliferation of spleen DCs^5^. Secondly, pDC lifespan is different among the different mouse strains, at least based on *in vitro* survival^7^,^8^. It remains to be demonstrated whether *in viv*o lifespan is also vastly different in mice with different genetic background. Thirdly, activation seems also to impact the lifespan of DCs, although the effects can be either positive or negative. It is known that activation during systemic viral infection or TLR ligand administration could result in rapid loss of pDCs, in an IFN-α-dependent manner that is partially dependent on the intrinsic apoptotic pathway^9^. On the other hand, *in vitro* activation with TLR ligand CpG can also significantly prolong survival of pDCs^10^.

Here we evaluate the *in vivo* lifespan of pDCs taking the above factors that influence pDC lifespan into consideration. Firstly, we compared BrdU labeling of pDCs in both the spleen and BM (where pDC differentiation occurs). Secondly, we evaluated pDC decay in the spleen and BM following 5-Fluorouracil (5-FU) treatment, which profoundly affects proliferating cells but not quiescent cells. We then compared the *in vivo* lifespan of pDCs from C57BL/6 and NZB mice that exhibit *in vitro* survival differences. Finally, we dissected cell death pathways regulating pDC survival, particularly upon activation. Overall, our results support that pDCs are short lived but their lifespan is influenced by genetic factors and activation.

## Results

### Differentiation of pDCs mainly occurs in bone marrow (BM)

**Figure 1:**
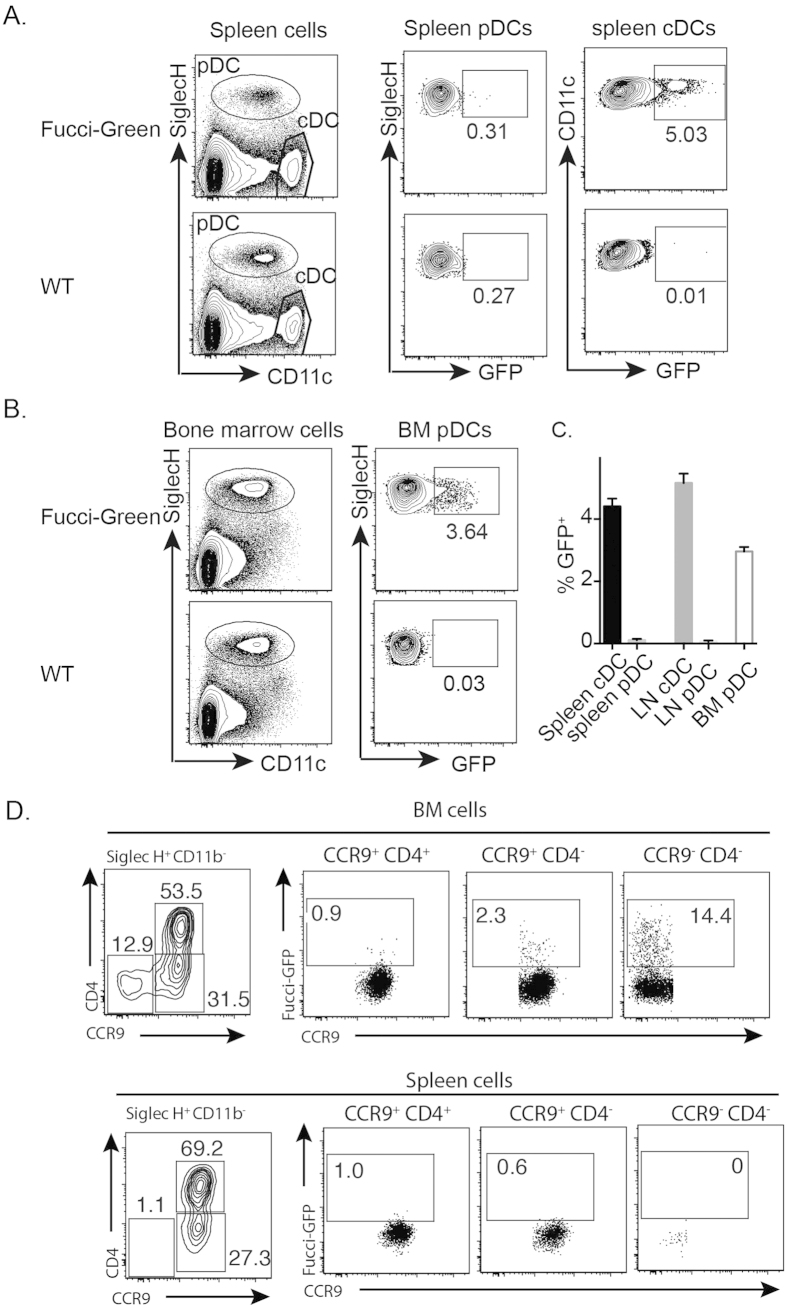
cDCs and pDCs are generated at different sites. Lymphoid organs were isolated from Fucci-Green mice and C57BL/6 mice. Cells were stained for surface markers. Gated cell populations were then analysed for expression of Geminin-GFP. (**A**) spleen cDCs and pDCs; (**B**) BM Siglec H^+^ cells. (**C**) Mean ± SEM of GFP^+^ DCs of spleen, LN and BM from 5 individual mice are shown for one of 3 independent experiments; all three experiments had similar results. (**D**) CD11b^−^Siglec H^+^ cells from BM and spleen of Fucci-Green mice were separated based on expression of CCR9 and CD4. The expression of Geminin-GFP by subsets was shown. More than 3 three experiments were performed.

A previous study using Ki-67 as a marker of proliferation found that 5% of splenic cDCs were Ki-67^+^ while only <0.5% of pDCs were Ki-67^+^, indicating that cDCs but not pDCs cycle in the spleen^5^. Fucci mice provide an elegant and facile way of visualizing live cycling cells by expression of Geminin-GFP^11^. Using Fucci mice, we confirmed the previous study using Ki-67, viz. that about 5% of cDCs and only <0.5% of pDCs in the spleen and LNs are GFP^+^ (Fig. 1). However, in the BM enriched for pDCs (but without an unequivocal cDC population), 3% of pDCs are GFP^+^ (Fig. 1). Based on expression of CD4 and CCR9, CD11b^−^Siglec H^+^ cells of BM cells contained three subsets: CD4^+^ CCR9^+^, CD4^−^CCR9^+^, and CD4^−^CCR9^−^ cells. GFP^+^ cells are mainly in CD4^−^CCR9^−^ cells while CD4^−^CCR9^+^ also contained a population of GFP^+^ cells. On the other hand, CD4^+^ CCR9^+^ cells contained very few GFP^+^ cells (Fig. 1D). In spleen, nearly all CD11b^−^Siglec H^+^ cells expressed CCR9 and did not express GFP (Fig. 1D). Overall, we concluded that proliferation of pDCs and cDCs occurs in different sites. Our data also predicts that splenic pDCs have a very limited capacity to take up BrdU *in situ*.

It remains to be resolved whether the presence of cycling CD11c^+^ DCs indicates that differentiated DCs can further divide^5^. We attempted to address this question using *in vitro* approaches. Firstly, CD11c^high^ (Siglec H^−^) spleen cells were purified and labeled with cell trace violet (CTV) dye, where dye dilution indicates cell division. Examination of DC (2 × 10^4^) cultured with or without 2 × 10^5^ feeder spleen cells revealed no conspicuous cell division in labeled DCs (Supplemental Fig. 1A). Secondly, *in vitro* culture of spleen cells from Fucci mice, in which live cycling cells express Geminin-GFP, permitted analysis of dividing DCs where no further recruitment of DC precursors could occur. In such a system, we observed a rapid loss of GFP^+^ cDCs (Supplemental Fig. 1B), indicating limited dividing capacity of cDCs, at least *in vitro*.

### Early BrdU labeling of BM pDCs is comparable to that of splenic cDCs

**Figure 2:**
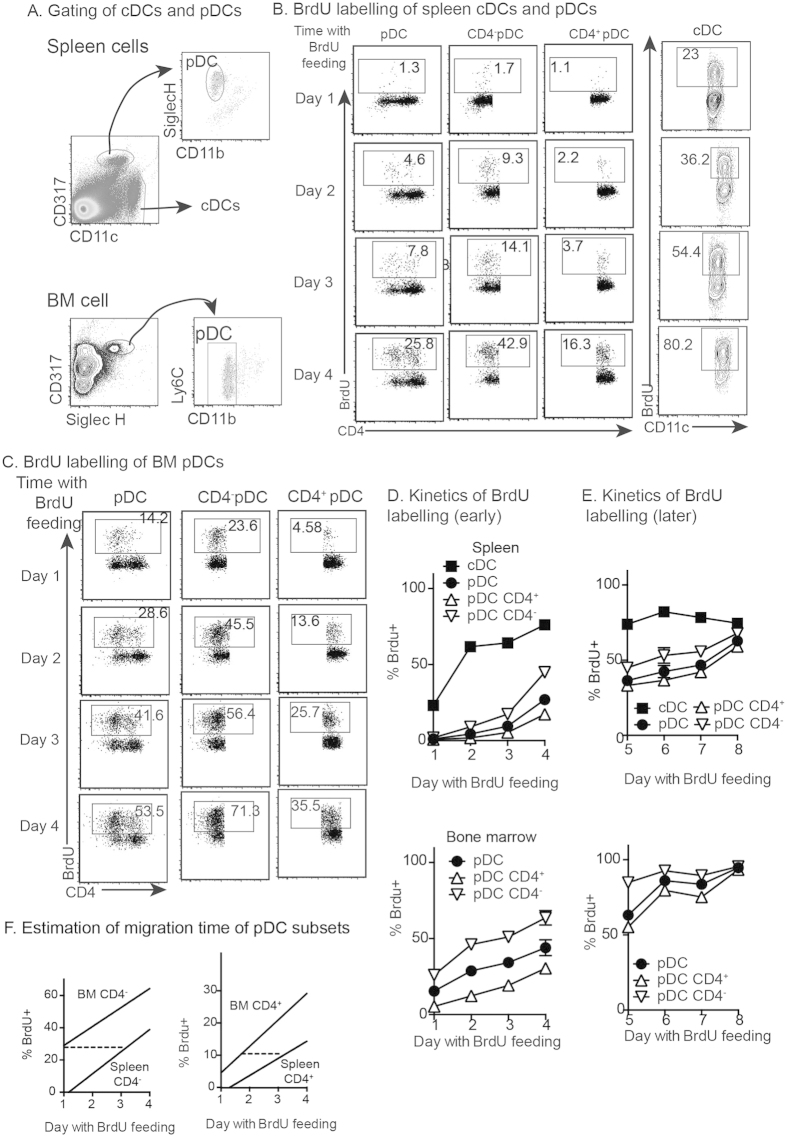
Early BrdU labeling occurs in BM pDCs and spleen cDCs. C57BL/6 mice were given an intraperitoneal injection of 0.1 mg BrdU and then fed with BrdU water (0.8 mg/mL). Spleens and BM were harvested at 1–4 days after BrdU exposure. Cells were stained for cell surface markers and then for intracellular BrdU. (**A**) Gating of pDCs and cDCs of spleen and BM. (**B**) Representative facs plots of BrdU labeling of gated spleen DC subsets. (**C**) Representative facs plots of BrdU labeling of BM pDCs. (**D**) Kinetics of BrdU labeling of DCs from spleen and BM. Two independent experiments were performed with similar results. (**E**) Estimation of migration time of pDC subsets, based on the data in (**D**).

Because of the difference in expansion site between splenic pDCs and cDCs, we reasoned that slower labeling of splenic pDCs by BrdU may not fully reflect longer lifespan of splenic pDCs. If the pDC lifespan is significantly longer than the cDC then BrdU labeling of BM pDCs should be significantly lower than splenic cDCs. To test this, we used a similar approach as previously reported^4^,^5^,^6^. BrdU was given as a single intraperitoneal injection of 1 mg and then maintained in the drinking water (0.8 mg/mL). Labeling of BrdU by DC subsets in spleens and BM was assessed at 1–4 days. The rate of BrdU labeling of pDCs and cDCs in the spleen was consistent with previous reports^4^,^5^,^6^. Splenic cDCs were 20% and >50% BrdU^+^ by days 1 and 3, respectively (Fig. 2A,B,D). In contrast, splenic pDCs were 1% and 6% BrdU^+^ by days 1 and 3, respectively (Fig. 2A,B,D). Similar to described previously^4^, labeling of splenic CD4^−^ pDCs was faster than that of more mature CD4^+^ pDCs. In BM, the rate of BrdU labeling of pDCs occurred much faster, so consequently BM pDCs were >10% and >40% BrdU^+^ by days 1 and 3, respectively (Fig. 2C,D). Among newly-derived (CD4^−^) pDCs (20–30% of Siglec H^+^ pDCs), the rate of BrdU labeling was even higher (Fig. 2C,D).

Notably, at day 4 after BrdU feeding, labeling of splenic pDCs, as well as BM pDCs, had not reached plateau. We therefore tested BrdU labelling for a further 4 days. In spleen, BrdU labeling of pDCs reached equivalent levels to cDCs by day 8 (Fig. 2E). In BM, BrdU labeling of pDCs plateaued by day 6 (Fig. 2E). Based on differential BrdU labelling of pDC subsets between BM and spleen (Fig. 2D), CD4^−^ pDCs take about 2 days to migrate from the bone marrow to the spleen, while CD4^+^ pDCs take ~1½ day (Fig. 2F).

Overall, results show that BrdU labeling of pDCs can occur as fast as that of cDCs. Hence, slower labeling of splenic pDCs may not directly indicate longer lifespan, rather reflect the migration time for BrdU^+^ pDCs to reach the spleen.

### Mature and immature pDCs can exit the bone marrow

**Figure 3:**
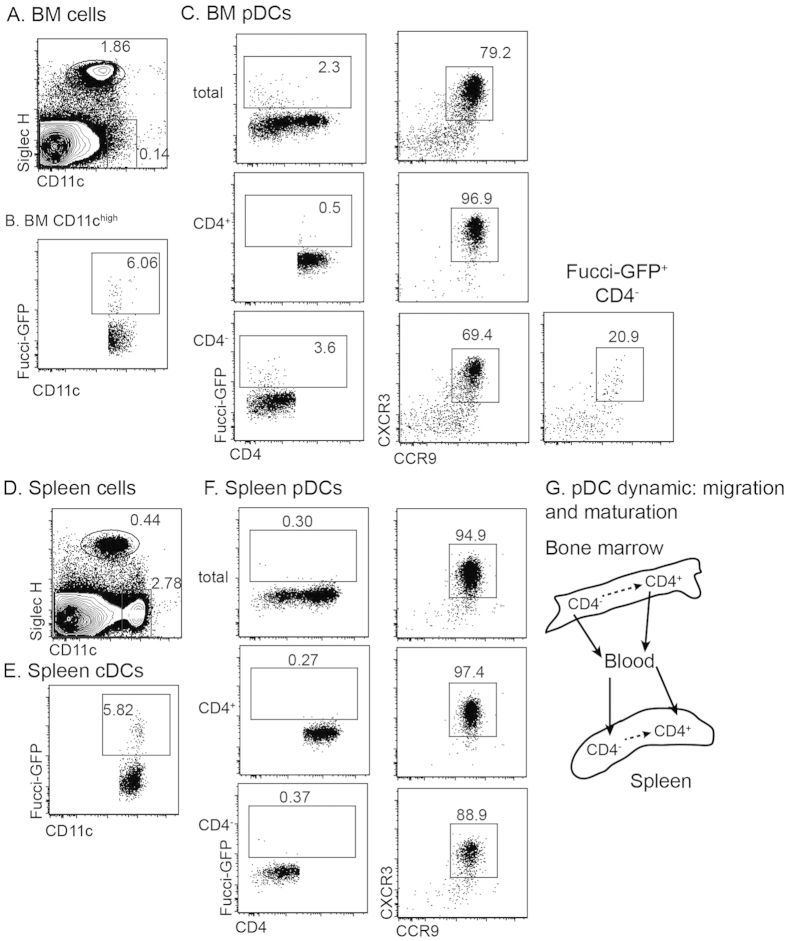
CD4^−^ and CD4^+^ pDCs exit bone marrow at a similar rate. Spleen cells and bone marrow cells were isolated from Fucci-Green mice. Cells were stained for surface markers. Gated cell populations were then analysed for expression of Geminin-GFP. (**A–C**) Bone marrow cells. (**D–F**) spleen cells. (**G**) Illustration of migrations and maturation of pDCs of bone marrow and spleen.

BrdU labeling indicates that pDCs are expanded in bone marrow but not in spleen. As both CD4^−^ (immature) and CD4^+^ (mature) pDCs exist in bone marrow, we tested the proliferative capacity of the two populations. Based on expression of Fucci-GFP, we revealed that only CD4^−^ pDCs had proliferative potential (Fig. 3A,C). Therefore, BrdU labelled CD4^+^ pDCs (Fig. 2) are likely to arise from the CD4^−^ fraction. Furthermore, CD4^+^ and CD4^−^ pDCs differed in expression of chemokine receptors: the majority of CD4^+^ pDCs co-express CXCR3 and CCR9, while the much reduced proportion of CD4^−^ pDCs, particularly Fucci-GFP^+^ CD4^−^ pDCs co-express CXCR3 and CCR9 (Fig. 3C). Despite the difference in maturation and chemokine receptor expression, both CD4^+^ and CD4^−^ pDCs exist in the spleen, albeit with an altered ratio (Fig. 3D,E). Furthermore, considering that BrdU^+^ CD4^+^ and CD4^−^ pDCs appeared in spleen in parallel, we suggest that both mature and immature pDCs exit the bone marrow. Thus, the pDC compartment in the periphery is a result of the continuous maturation from CD4^−^ to CD4^+^ and migration of both CD4^+^ and CD4^−^ pDCs from the bone marrow (Fig. 3G). Of note, splenic cDCs and BM CD11c^high^ cells had similar proportions of Fucci-GFP^+^ cells (Fig. 3B,E).

### Splenic pDC and cDC numbers decay at a similar rate

To ascertain the lifespan of pDCs further, we followed the decay of pDCs upon blockade of proliferation of pDCs and their immediate precursors. To this end, we explored the unique property of a pyrimidine analogue 5-FU that prevents normal DNA replication and thus has profound effects on proliferating cells but not on quiescent cells. We used a similar regimen to that adopted for *in vivo* enrichment of hematopoietic stem cells^12^.

**Figure 4:**
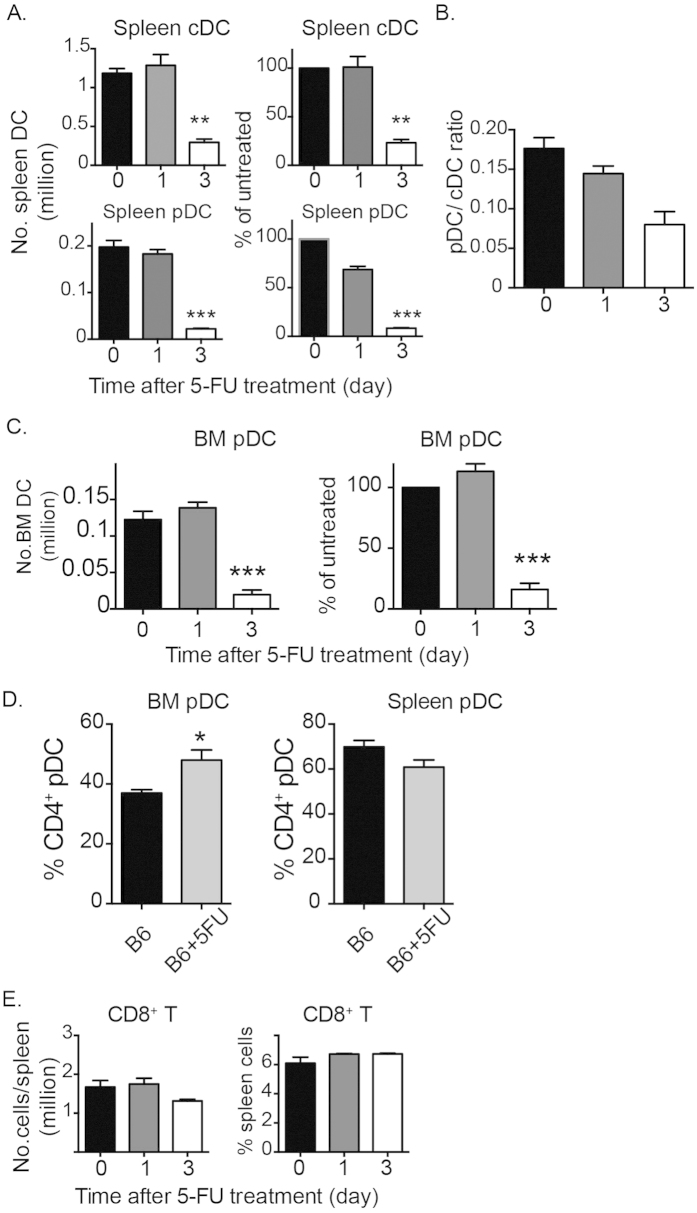
Spleen cDCs and pDCs decay similarly following 5-FU treatment. C57BL/6 mice were given a single dose of 5-FU intravenously. DC composition in spleen and BM was then evaluated at 1 day and 3 days after treatment. (**A**) Mean ± SEM of numbers and percentages of spleen DCs (**B**) ratio of spleen pDCs to cDCs. (**C**) Mean ± SEM of numbers and percentages of BM pDCs. (**D**) percentages of CD4^+^ pDCs in BM and spleen with or without 5-FU treatment for 3 days. (**E**) Mean ± SEM of numbers and percentages of long-lived spleen CD8^+^ T cells are shown. Data (**A–E**) are from 6 untreated mice and 3 treated mice for each time point from one of 3 independent experiments; *P < 0.05, **P < 0.01, ***P < 0.001 by Student t test, compared to untreated.

Mice were treated with a single dose of 5-FU (150 mg/kg body weight, intravenous injection), and both cDCs and pDCs were enumerated 1–3 days after injection. At 1 day after 5-FU administration, loss of cDCs and pDCs in the spleen was very minimal. At 3 days after 5-FU administration, there were profound losses of both cDCs and pDCs in the spleen (Fig. 4A). Of note, the ratios of pDCs to cDCs were not significantly altered over the course of the experiment, indicating a similar loss of splenic pDCs and cDCs (Fig. 4B). We also observed that profound loss of BM pDCs occurred at 3 days after 5-FU treatment (Fig. 4C). Given that pDCs can be divided into two subpopulations based on CD4 expression, we compared the loss of two subsets after 5-FU treatment. Although both subsets were reduced after 5-FU treatment, percentage of CD4^+^ pDCs in BM cells was significantly increased at 3 days after 5-FU treatment while percentage of CD4^+^ pDCs in spleen cells was not (Fig. 4D).

We found that 5-FU treatment had no overt effect on long-lived CD8^+^ T cells (Fig. 4E), suggesting that loss of DCs is unlikely due to 5-FU directly inducing apoptosis. Thus, loss of differentiated pDCs and cDCs is comparable between the two subsets.

### *In vivo* lifespan of pDCs differs between C57BL/6 and NZB mice

**Figure 5:**
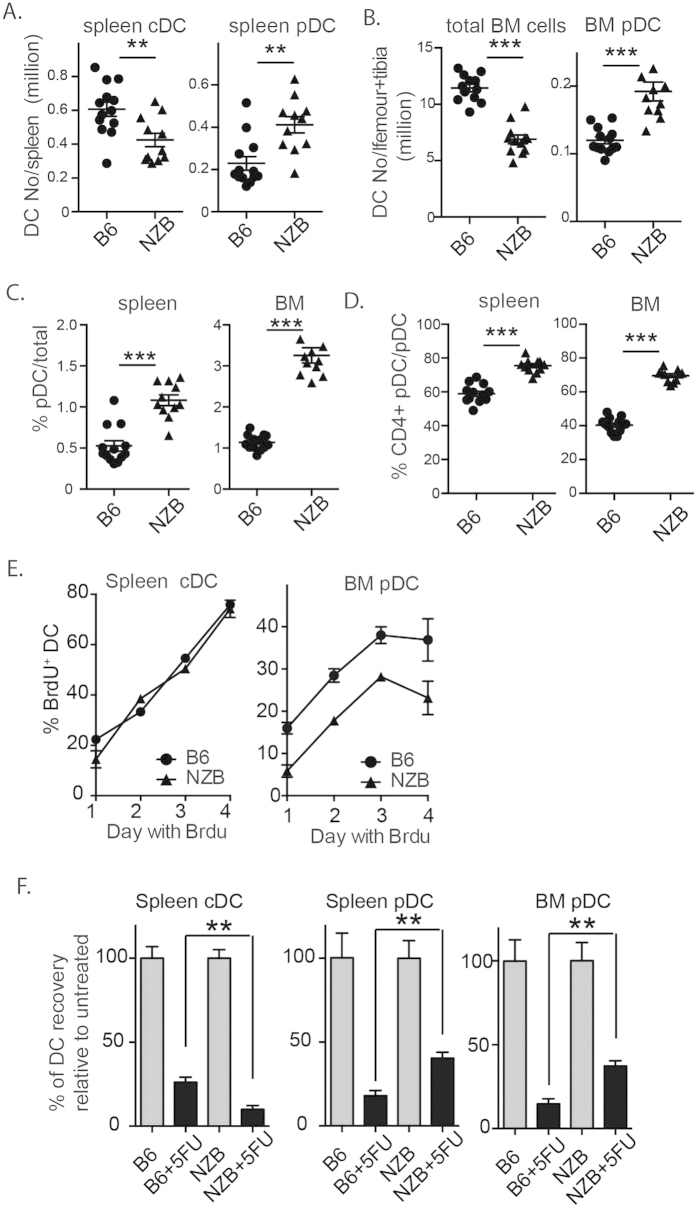
pDCs of NZB mice survive longer than pDCs of C57BL/6 mice *in vivo*. (**A–D**) Spleens and BM of age- (6–8 weeks old) and sex-matched C57BL/6 and NZB mice were harvested. Single cell suspension was stained for DC surface markers. Mean ± SEM of numbers and proportions of total and gated populations are shown for 13 C57BL/6 and 11 NZB mice pooled from 2 experiments; **P < 0.01, ***P < 0.001 by Student t test. (**E**) C57BL/6 and NZB mice were fed BrdU water. Organs were harvested at different days for evaluation of BrdU labeling of gated DC populations. (**F**) C57BL/6 mice and NZB mice were given a single dose of 5-FU intravenously. After 3 days, DC composition in spleen and BM was evaluated. Mean ± SEM of 3 mice from each strain are shown for one of three independent experiments. **P < 0.01 by Student t test.

pDC survival is different among different mouse strains, at least based on *in vitro* assays^7^,^10^. In our hands, splenic DCs of lupus prone NZB mice survived better than pDCs of C57BL/6 mice over 3 days in culture. Here we aimed to compare the *in vivo* lifespan of pDCs in the two strains of mice. In a sex/age-matched cohort (6–8 weeks old females), we observed that splenic pDCs and cDCs, were respectively, 100% more and 50% less abundant in NZB mice than in C57BL/6 mice (Fig. 5A). Despite significantly lower total BM cells in NZB mice, pDCs in the BM were 70% more abundant (Fig. 5B). Proportionally, pDCs were also significantly more abundant in the spleen and BM of NZB mice than in C57BL/6 mice (Fig. 5C). Furthermore, when pDCs were segregated into CD4^−^ (less matured) and CD4^+^ (more matured, derived from CD4^−^ pDCs) subpopulations, we found that NZB pDCs have more CD4^+^ pDCs (BM CD4^+^ pDCs C57BL/6 50% vs NZB > 70%; splenic CD4^+^ pDCs C57BL/6 60% vs. NZB > 80%) (Fig. 5D), indicating that NZB pDCs may have a longer *in vivo* lifespan.

In addition, we also investigated the expression of Ly49Q by pDCs from the two types of mice. Interestingly, in both BM and spleen, we did not observe significant differences in Ly49Q (Supplemental Fig. 2). Furthermore, to exclude the possibility of increased proliferation by NZB pDCs being responsible for increased numbers of pDCs, we compared pDCs from young and old C57BL/6 mice and NZB/W mice for expression of Ki67, a cellular marker for proliferation. Spleen pDCs from all mice did not contain a clear population of Ki67^+^ cells (Supplemental Fig. 3A) while spleen cDCs from all mice contained a Ki67^+^ population (Supplemental Fig. 3B). Within BM SiglecH^+^ cells, a similar population of Ki67^+^ cells (mainly CD4^−^) existed in all mice (Supplemental Fig. 3C). Thus, the difference in pDCs between C57BL/6 mice and NZB/W mice is unlikely due to expansion/proliferation.

Next we tested whether BrdU labeling can reveal the difference in cell lifespan, particularly BM pDCs, between C57BL/6 and NZB mice. We did detect lower BrdU labeling of NZB pDCs, compared to C57BL/6 pDCs (Fig. 5E). On the other hand, BrdU labelling of splenic cDCs was very comparable between C57BL/6 and NZB mice. Thus, BrdU labelling does indicate that NZB pDCs might have an increased lifespan.

Finally, we compared the DC lifespan of these two strains of mice by following DC decay after a single dose of 5-FU treatment. By 3 days after 5-FU treatment, both cDCs and pDCs from the two strains of mice were reduced to 10–30% of untreated. Of note, the reduction of pDCs from the spleen and BM of NZB mice was significantly less than that of C57BL/6 mice, while decay of splenic cDCs was faster in NZB mice (Fig. 5F). As we have previously shown that BCL-2 over-expression enhanced the survival of pDCs but not cDCs *in vitro*^10^,^13^, we tested the effect of 5-FU treatment on DC decay in BCL-2 tg mice. Increased BCL-2 expression prevented the loss of pDCs at 3 days after 5-FU treatment but did not prevent loss of cDCs (Supplemental Fig. 4A). Of note, Spleen CD4^+^ pDCs are proportionally increased in BCl-2 tg mice, compared to C57BL/6 mice. Overall, pDCs of NZB mice have a demonstrable longer lifespan than pDCs of C57BL/6 mice *in vivo*.

### Survival of activated pDCs is subjected to regulation by multiple pathways

We showed recently that resting pDCs are largely dependent on BCL-2 and MCL-1 for survival^8^. How activation affects the pDC survival program remains less defined. One study reported that activation during systemic viral infection and TLR agonism resulted in rapid loss of pDCs^9^. Other studies, albeit *in vitro*, seem contradictory: TLR ligation up-regulated pro-survival molecules and enhanced pDC survival^4^,^10^,^14^. Quantification of pDCs following *in vivo* activation can be confounded by sequestration into inflamed tissues^15^ and by identification due to down-regulation of pDC markers following activation^16^. Therefore, we initially purified pDCs to investigate their *in vitro* survival.

**Figure 6:**
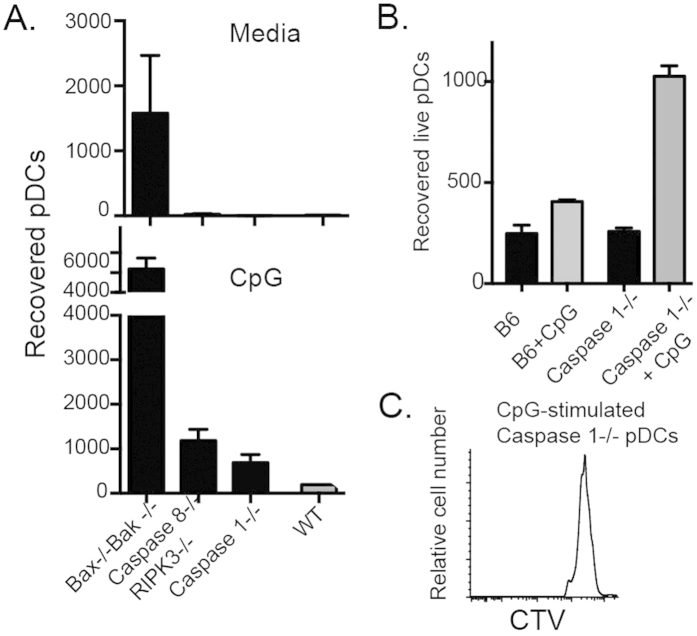
pDC survival is differentially affected by different death pathways. (**A**) Spleen pDCs were purified from WT, Bax−/−Bak−/bone marrow chimera, Caspase 8−/−/RIPK3−/− and Caspase 1−/− mice. pDCs were cultured at 10^4^ cells/well with or without 20 nM CpG for 2 days. Live pDCs were enumerated. Histograms represent the mean ± SEM of viable DCs of each group. Arrows indicate comparison between WT with or without CpG. (**B,C**) pDCs were purified from C57BL/6 mice and Caspase 1−/− mice. Purified pDCs were labeled with CTV dye. Labeled DCs (10^4^/well) were then cultured with 10^5^ unfractionated spleen cells. Cultures were either with or without 20 nM CpG for 24 hrs. Viable DCs were then enumerated. Bar graphs (**B**) show the means numbers and SEM of triplicate pDC cultures. (**C**) Histograms show the CTV labeling of Caspase 1−/− pDCs. Three similar experiments were performed.

Cell survival/death is regulated by multiple pathways including intrinsic and extrinsic apoptosis, pyroptosis and necroptosis^17^. To investigate the contribution of the different pathways, we assembled the following mouse models: Bax/Bax−/− (defective in BCL-2 regulated apoptosis), Caspase 8/RIPK3−/− mice (defective in death-receptor-regulated apoptosis and necroptosis), Caspase 1−/− (defective in pyroptosis). Purified pDCs from these models were analysed for survival. As previously reported^13^, isolated pDCs from WT (C57BL/6) mice die rapidly in culture (Fig. 6A). Among pDCs with different defects in cell death, Bax/Bak deficiency greatly enhanced pDC survival while deficiency in other pathways had either no or minor impact on survival of unstimulated pDCs (Fig. 6A). Activation of pDCs with CpG1826 enhanced pDC survival in all groups (Fig. 6A). Deficiency in either Caspase 1 or Caspase 8/RIPK3 augmented the survival of activated pDCs, (unlike unstimulated pDCs) (Fig. 6A). To test whether other spleen cells would impact pDC survival, we cultured CTV-labelled pDCs of WT or Caspase 1−/− mice with unfractionated WT spleen cells. We observed that enhanced survival of Caspase 1−/− pDCs was further increased by the presence of spleen cells (Fig. 6B). Caspase 1−/− pDCs did not proliferate in culture (Fig. 6C) and therefore increased recovery likely resulted from increased survival. The *in vitro* findings suggest that survival of activated pDCs is regulated by several pathways, whereas resting state pDCs use predominantly the mitochondrial BCL-2 pathway.

## Discussion

Estimation of the pDC lifespan by BrdU labeling in earlier studies suggested that pDCs, relative to lymphoid resident cDCs, are long-lived^4^,^5^,^6^. However, estimation of pDC lifespan by parabiosis experiments, on the other hand, suggest that the lifespan of pDCs is short, even shorter than cDCs^5^. Ample evidence from *in vitro* experiments also suggests that pDCs are short-lived^13^. How these discrepancies can be reconciled has not been properly addressed. Here we reappraised the lifespan of pDCs. We conclude that splenic pDCs are generally short-lived, but that the pDC lifespan is subjected to regulation by multiple mechanisms.

BrdU labeling has been a common method for estimation of lifespan of various cell types including T cells^18^ and dendritic cells^2^. With the exception of one report^19^, most such studies found that splenic pDCs had a slower rate of BrdU labeling than cDCs^4^,^5^,^6^. This slower rate of BrdU labelling was interpreted as pDCs having a longer lifespan than cDCs. However, it has become evident that splenic cDCs result from expansion from pre-DCs which have migrated from the BM^20^. About 5% of spleen cDCs express the proliferation marker Ki-67 at any given time^5^. On the other hand, very few spleen pDCs express Ki-67. Using Fucci-GFP to track cells in cycle, we also found that there were very few cycling pDCs in the spleen. Instead, 3% of BM pDCs (defined as SiglecH^+^ CD11b^−^) express GFP in Fucci-GFP mice, suggesting that pDC differentiation/expansion occurs mainly in the BM. Thus, BrdU labeling of pDCs does not occur in the spleen, rather BrdU^+^ pDCs in the spleen represent migrated cells after BrdU labeling in the BM. Therefore, the slower appearance of BrdU labeled cells in the spleen is also attributable to the migration time from the BM. By comparing the kinetic of BrdU labelling of BM pDCs and spleen pDCs, we suggest that migration times are about 2 days for CD4^−^ pDCs and slightly less for CD4^+^ pDCs.

Parabiosis experiments previously performed showed rapid disappearance of parabiont-derived DCs after severing parabiosis, suggesting that the lifespan of pDCs was short^5^. However, the parabiosis system has some caveats: firstly, reconstitution never reaches equilibrium for parabiont DCs^5^; secondly, surgical separation of parabionts may have differential consequences on resident cDCs and circulating pDCs. We attempted to address this with a different approach by investigating the decay of pDC numbers by stopping the “new supply” of DCs via 5-FU treatment. 5-FU has profound effects on proliferating cells but not on quiescent cells. Germanely, it has been reported that human DCs are resistant to 5-FU *in vitro*^21^. After 5-FU treatment, we found that the kinetics of splenic pDCs and cDCs was similar: no clear loss at 1 day after treatment, >70% loss by 3 days after treatment. Thus, tracking DC “decay” also supports that pDCs are most likely short-lived.

In mouse models of lupus, deletion or impairment of pDCs ameliorates lupus disease^22^,^23^. These recent studies had provided direct evidence of pDC involvement in development of autoimmune diseases like lupus. Notably, there are discernable increases in pDC numbers in the lupus-prone NZB mouse strain^7^,^10^. Whereas increases in pDC numbers could reflect increased production of these cells, it could also be caused by enhanced survival. At least *in vitro*, survival of pDCs from NZB mice is prolonged compared with that from C57BL/6 mice^7^,^10^. Our current study provides some evidence that NZB pDCs might have a longer lifespan than C57BL/6 pDCs *in vivo*. Firstly, based on the maturation marker CD4, the spleen and BM of NZB mice harbours significantly higher proportions and numbers of more mature (CD4^+^) pDCs than that of C57BL/6 mice. Notably, although pDC compartment is also increased in 129sv mice^24^, we found that CD4^+^ pDCs was not proportionally increased in 129sv mice (Supplemental Fig. 4B). It suggests that not all strains of mice control pDC compartment by the same way. It can be due to mechanisms other than cell survival like differentiation and expansion/proliferation. Nevertheless, the difference in pDC compartment between C57B/6 and NZB mice is unlikely to be due to pDC proliferation/expansion in the periphery since there was no Ki67^+^ pDCs in spleens of both young and old C57BL/6 and NZB (or NZB/W) mice. Furthermore, Ki67^+^ pDCs in BM was comparable between C57BL/6 and NZB mice. Secondly, decay of splenic pDCs after 5-FU treatment was significantly slower in NZB mice than C57BL/6 mice. As BrdU labeling is very modest in splenic pDCs (and has the caveat of migration from BM), we compared BrdU labeling of pDCs in BM from C57BL/6 and NZB mice and found the labeling in NZB mice to be slower, again indicating longer survival. The overall data support that NZB pDCs have a longer lifespan than C57BL/6 pDCs. Despite evidence that pDCs are critically involved in disease development of lupus prone mice^22^,^23^, it is currently unclear whether prolonged survival of pDCs in lupus-prone strain contributes to development of autoimmune diseases. In addition, it is also unclear about the molecular basis of this enhanced survival. Given that BCL-2 regulated apoptosis is critical for pDC survival^8^,^10^, dysregulation in this death pathway may likely contribute to increased survival. Of note, in contrast to survival differences of pDCs, cDCs from NZB mice and C57BL/6 mice had similar survival times *in vivo* in our study, reinforcing our notion that survival of pDCs and cDCs have different regulatory mechanisms^8^.

In unstimulated cultures, survival of pDCs is largely BCL-2 regulated. Survival of pDCs from BCL-2 transgenic mice are significantly prolonged^10^,^13^. On the other hand, the BCL-2 antagonist, ABT-199 drastically reduces the viability of pDCs^8^,^10^. Here we extended these investigations by examining the contribution of other cell death mechanisms. As expected deficiency in Bax and Bak greatly enhanced pDC survival, whilst perturbation of other caspases, including caspase-1, and −8 had minimal impact on pDC survival. Similarly, a defect in RIPK3, a kinase critical for necroptosis, also minimally impacted pDC survival. Thus, for unstimulated pDCs, survival is largely controlled by the BCL-2 apoptotic pathway.

Once activated, pDC survival is more complex. Two opposing forces can be experimentally demonstrated. On one hand, TLR-stimulated pDCs upregulate anti-apoptotic molecules and show enhanced survival^4^,^8^,^10^,^14^. This enhanced survival after stimulation has been proposed to be a mechanism responsible for drug resistance in lupus treatment^25^. On the other hand, the mechanism enhancing activation-induced cell death also operated simultaneously. It is evident from the current study that Caspase-1-mediated pyroptosis, Caspase-8/RIPK3-mediated apoptosis/necroptosis plays a role in the death of activated pDCs. As a consequence, pDCs from mice defective in these molecules showed enhanced survival beyond activated WT pDCs. It is likely that multiple death pathways operate in such a situation. It remains uncertain whether different activating ligands may elicit a predominant pathway and determine the outcome (survival or death).

Despite *in vitro* data showing that activation enhances pDC survival and that blocking multiple death mechanisms further enhances this survival, *in vivo* loss of pDCs is very profound following systemic activation when death pathways are perturbed. The contrast findings of *in vitro* and *in vivo* systems remain to be reconciled. A possibility is that pDCs following *in vivo* activation can be migrated into inflamed tissues^15^. Loss of pDCs in lymphoid organs may not necessarily equal to cell death. Reduction in pDCs following infection can also be due to production of pDCs. Inflammation-induced cytokines like GM-CSF can alter the homeostasis of DC subsets and reduce pDC differentiation^26^. These possibilities remain to be explored.

It has been reported that about 5% of splenic cDCs express the proliferation marker Ki-67 at any given time^5^. We detected a similar proportion of cycling CD11c^high^ spleen cells in Fucci-GFP mice. The question is whether these cells reflect proliferative potential of fully differentiated cDCs or a transit stage of newly differentiated cDCs from pre-DCs. At least *in vitro*, CTV-labeled cDCs did not show proliferation. Furthermore, spleen cells from Fucci-GFP mice lose most of the GFP^+^ cDCs within 20 hours in culture. Thus, cDCs per se may have very limited potential for proliferation. It is unclear whether cDCs behave similarly *in vivo*, since the direct tracking of DC fate *in vivo* remains challenging.

Identification of pDCs by flow cytometry is largely based on expression of particular surface markers, such as CD45RA, BST2 (CD317) and Siglec H. Although CD317 is a good marker of pDCs in the naïve mice, it is up-regulated on many cell types under inflammatory conditions^27^,^28^. Siglec H is therefore a more specific marker for pDCs^29^,^30^. In most of our studies, we identified pDCs as CD11c^int/low^, CD11b^−^, Siglec H^+^. Additional markers including CD317, CD45RA, Ly6C, CD4, CD8 and CCR9 were also used to confirm the identity of pDCs. Isolated Siglec H^+^ spleen cells showed uniform sensitivity to the BCL-2 antagonist BH3 mimetic ABT-199 while cDCs and other myeloid cells were resistant to ABT-199^8^. There is more heterogeneity among Siglec H^+^ CD11b^−^ cells in BM. Although most of the pDCs are CCR9^+^, about 10% of Siglec H^+^ CD11b^−^ BM cells are CCR9^−^CD4^−^, which is a negligible population within the spleen Siglec H^+^ cells. This BM CCR9^−^CD4^−^ population contained 14% GFP^+^ cells. They are likely the precursors for both pDCs and certain cDCs^31^. Within CCR9^+^ cells, GFP^+^ cells are within the CD4^−^ cohort. However, heterogeneity of Siglec H^+^ CD11b^−^ BM cells should have limited impact on Siglec H as a robust marker for pDC identification, particularly for cells from unperturbed mice.

Taken together, splenic pDCs of C57BL/6 mice might have a shorter lifespan than previously estimated and be more closely aligned with the parabiotic studies. This reappraisal partially resolves the discrepancy of *in vitro* and *in vivo* lifespan data of C57BL/6 pDCs. Nevertheless, the pDC life span can be significantly affected by genetic factors and activation status. As regulation of pDC survival is an integral part of pDC homeostasis, further insight into how pDC survival is regulated will enhance our understanding of the physiological and pathological importance of pDCs.

## Methods

### Mice

C57BL/6 (B6, wt), NZB and NZB/W F1 mice, Fucci-GFP^11^ and Caspase 1−/− mice^32^, and Caspase 8/RIPK3−/− mice^33^ were housed under specific pathogen-free conditions at The Walter & Eliza Hall Institute of Medical Research. Bax−/−Bak−/− mice are lethally irradiated Ly5.1 mice reconstituted with Bax−/−Bak−/− fetal liver cells. All experiments were performed in accordance with relevant guidelines and regulations that were approved by the The Walter & Eliza Hall Institute of Medical Research animal ethics committee (Project #2013.015, #2014.023).

### Cell preparation, antibodies and flow cytometry

Cells were prepared from spleen and pooled subcutaneous lymph nodes (inguinal, axial, brachial, cervical) unless specified, and were prepared by digestion in collagenase/DNAse 1 as described^34^. Antibodies (Ab) used in this study were anti-CD11c (HL3, APC, APC-Cy7), CD11b (M1/70, BV421, PE-Cy7), CD8 (53–6.7, APC-Cy7), CD4 (RM4-4, APC, Pacific Blue), CCR9 (CW-1.2, PE-Cy7), CXCR3 (CXCR3-173, FITC), I-A/I-E (M5/114.15.2, FITC, PE-Cy7), Ly6C (AL-21, APC-Cy7, FITC, PE-Cy7), Siglec H (eBio440c, PE) (ebioscience, San Diego, CA) and CD317 (eBio927, APC) (ebioscience). All Abs were purchased from BD Biosciences except where stated otherwise, with cell numbers determined by the addition of fluorochrome-conjugated calibration beads (BD Biosciences) directly to the samples. Data were collected using a FACScanto or FACSverse (BD Biosciences) and analyzed using FlowJo software (Treestar, Ashland, OR). Cell sorting was performed using a FACSaria or an Influx cell sorter (BD Biosciences, San Jose, CA).

### BrdU labeling

For continuous BrdU labeling, groups of 3–4 mice were initially injected intraperitoneally with 0.1 mg bromodeoxyuridine (BrdU) (Sigma, St Louis, MO) in saline and then continuously given BrdU (0.8 mg/mL) in drinking water. After various times, lymphoid organs were harvested and cell suspensions prepared.

BrdU staining was then performed with BD BrdU staining kit following the manufacturer’s instruction.

### 5-FU treatment

Mice were injected intravenously with a single dose of 150 mg/kg 5-FU intravenously^12^. Mice were killed 1–4 days after injection. Cell suspensions of spleens, BM and peripheral lymph nodes were prepared and stained for DC surface markers.

### Ki67 staining

Spleen cells and BM cells were firstly stained for cell surface markers of dendritic cells. Fixed and stained cells were then stained for expression of Ki67 with anti-human Ki67 antibody (clone B56, BD Pharmingen).

### DC survival assays and DC activation

Spleen cells were enriched for DCs using a Nycodenz density gradient as previously described^34^. Enriched cells were further purified on FACS sorters. Purified DCs with or without CTV (Invitrogen, ThermoFisher, Waltham, MA) labeling (as per manufacturer’s protocol) were cultured at 1–2 × 10^4^ in 200 μL RPMI supplemented with 10% FCS in flat-bottom 96-well plates. In some experiments, unfractionated Ly5.1 spleen cells were included in cultures as feeder cells. Cells were stimulated with or without 20 nM CpG1826 (Sigma). Upon harvesting, cells were stained for cell surface markers. Cell survival was measured by flow cytometry with PE or APC-conjugated FACS calibration beads (BD Biosciences) and PI to determine the number of viable cells.

### Western blot analysis

Cell preparation, protein extraction and Western blot were performed as previously described^10^. Briefly, cells were collected, washed once with mouse-tonicity PBS, then frozen immediately on dry ice, and stored at −80 °C before analysis.

### Statistical analysis

Statistical comparisons of mean difference between two groups from independent experiments were made using a two-tailed Student’s t test with Prism v.5.0 software (GraphPad, San Diego, CA). P values < 0.05 were considered statistically significant.

## Additional Information

**How to cite this article**: Zhan, Y. *et al.* Plasmacytoid dendritic cells are short-lived: reappraising the influence of migration, genetic factors and activation on estimation of lifespan. *Sci. Rep.* **6**, 25060; doi: 10.1038/srep25060 (2016).

## Supplementary Material

Supplementary Information
