# Supplementary material for: Plasmacytoid dendritic cells are short-lived: reappraising the influence of migration, genetic factors and activation on estimation of lifespan
Source: Sci Rep. 2016 Apr 26;6:25060. doi: 10.1038/srep25060 (PMC4844974; doi:10.1038/srep25060)
Supplement: Supplementary Information [file srep25060-s1.pdf]

Plasmacytoid dendritic cells are short-lived: reappraising the influence of migration, genetic factors and activation on estimation of lifespan

Yifan Zhan, Kevin V Chow, Priscilla Soo, Zhen Xu, Jamie L Brady, Kate E Lawlor, Seth L Masters, Meredith O'keeffe, Ken Shortman, Jian-Guo Zhang, and Andrew M Lew

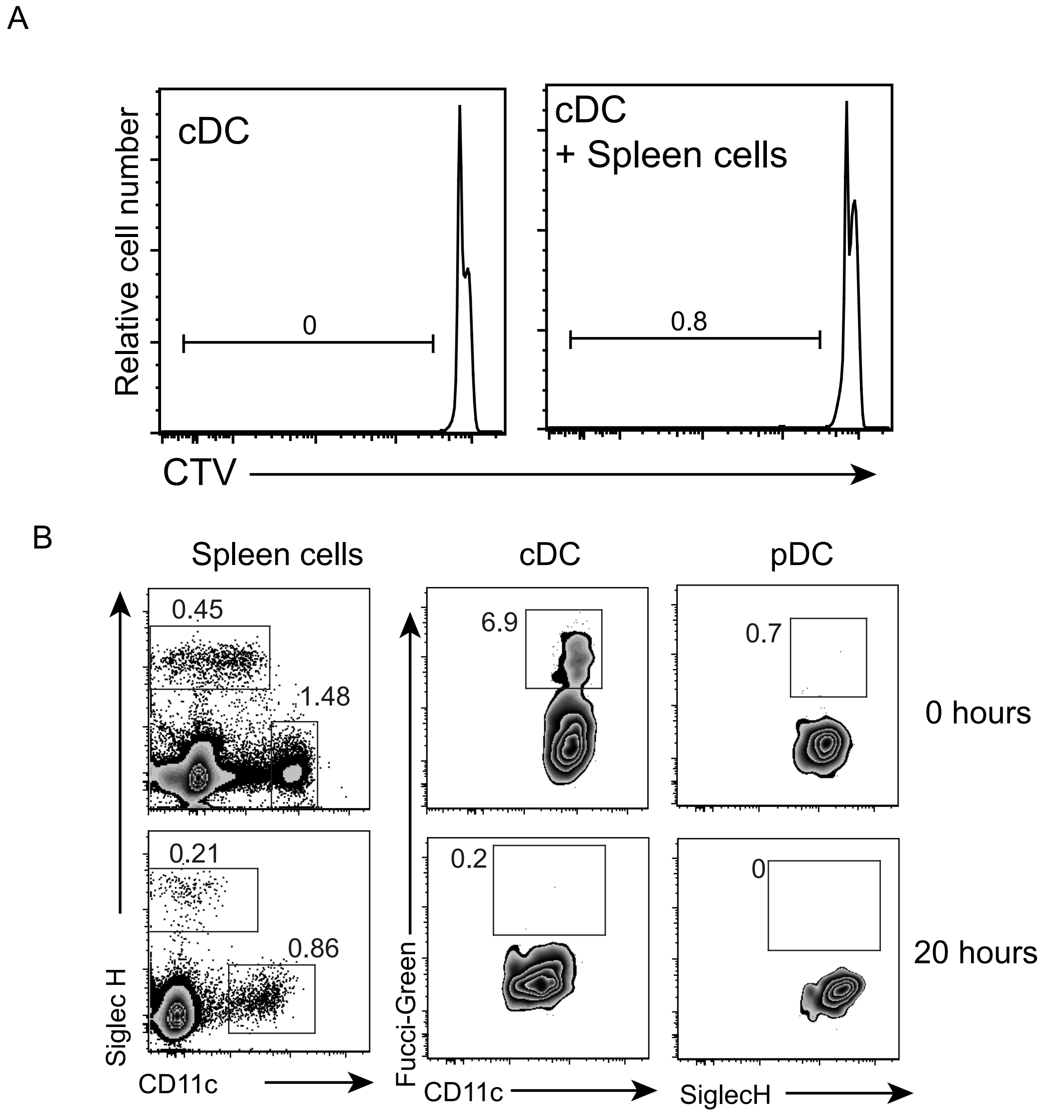

SF1. Differentiated spleen cDCs have limited potential to proliferate.

A. Purified cDCs were labeled with CTV dye. Labeled DCs (10^4/well) were then cultured with or without 10^5 unfractionated spleen cells as feeders. Histograms show the CTV labeling of cDCs after 48 hrs in culture.

B. Spleen cells from Fucci-GFP mice were cultured. Gated cDCs and pDCs before culture or after culture for 20 hrs are shown for GFP intensity.

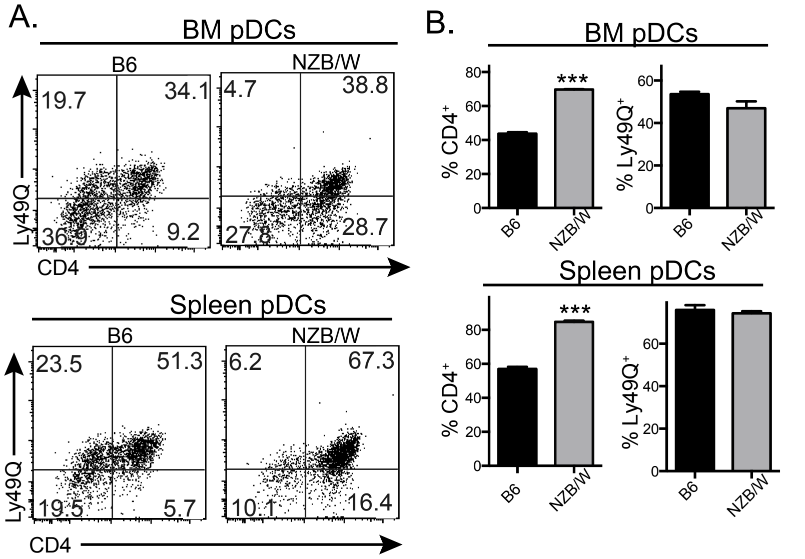

SF2. Expression of CD4 and Ly49Q by pDCs. Spleen and BM were harvested C57BL/6 and NZB/W mice (female, 8 week old). Cells were stained for DC surface markers. Gated DC populations are shown for Ki67 expression.

(A) FACS plts show the expression of CD4 and Ly49Q by pDCs of BM and spleen.

(B) Bar graphs show the percentage of $CD4^+$ and $Ly49Q^+$ pDCs of BM and spleen.

***P<0.01, compared to B6 mice by T test. Three independent experiments were performed.

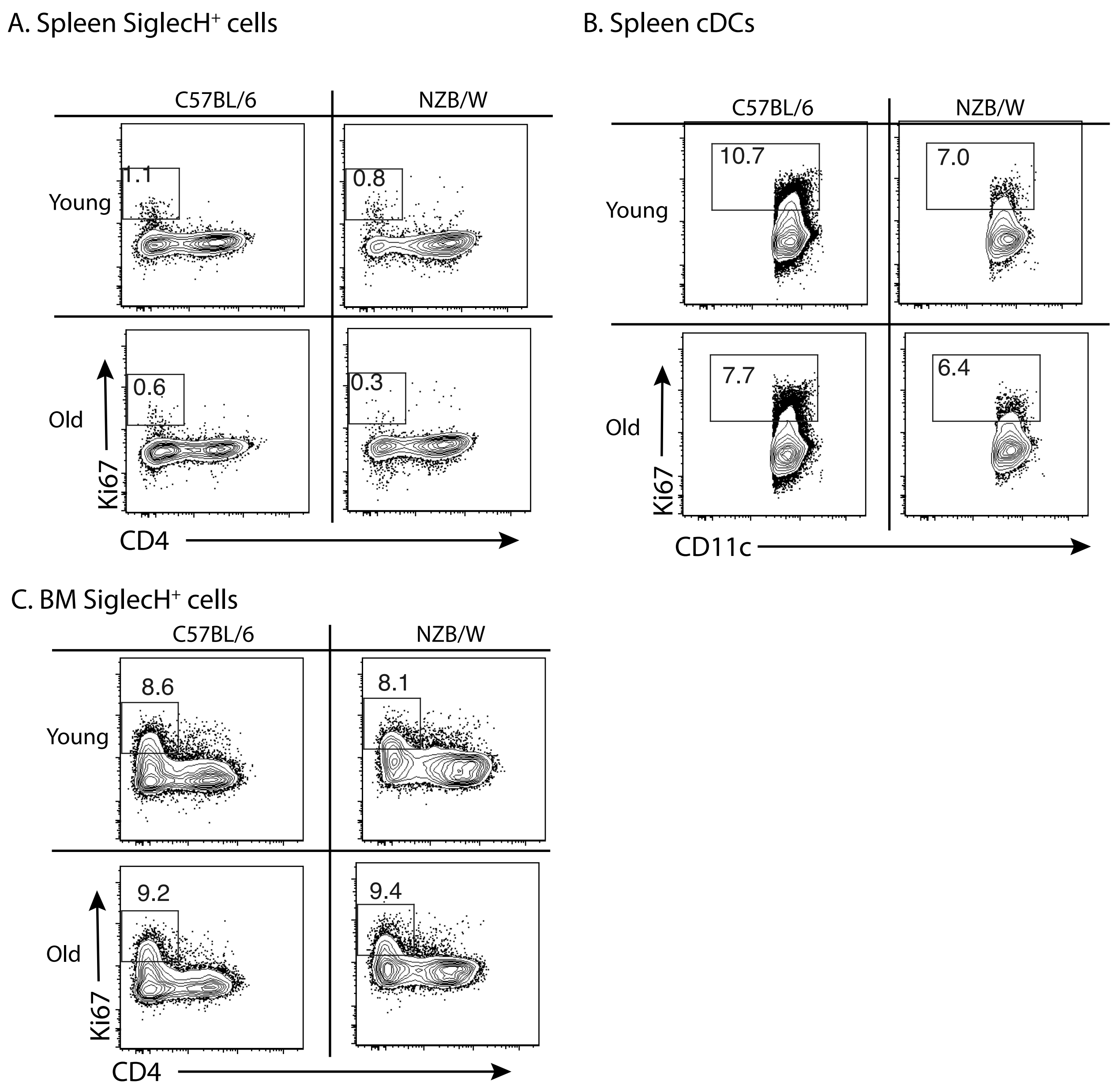

SF3. Both spleen pDCs of C57BL/6 and NZB/W mice did not proliferate. Spleen and BM were harvested from old (28 week old) and young (8 week old) of C57BL/6 and NZB/W mice. Cells were stained for DC surface markers and then intracellular Ki67. Gated DC populations are shown for Ki67 expression. (A) spleen SiglecH^{+} cells, (B) spleen cDCs, and (C) BM SiglecH^{+} cells. Three independent experiments were performed.

A.

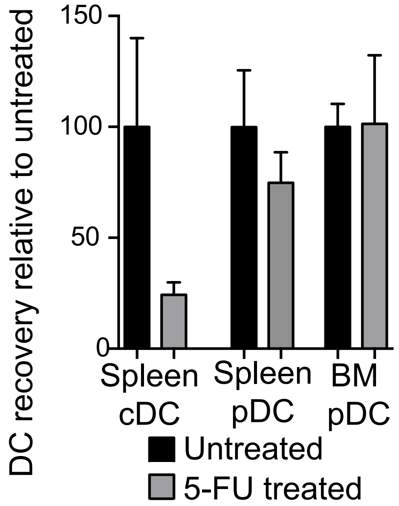

SF4A. pDCs of BCL-2 transgenic mice have prolonged in vivo survival.
BCL-2 transgenic mice were given a single dose of 5-FU intravenously.
DC composition in spleen and BM was then evaluated 3 days after treatment.
Histogram represent % of DC recovery relative to untreated BCL-2 transgenic mice.

B.

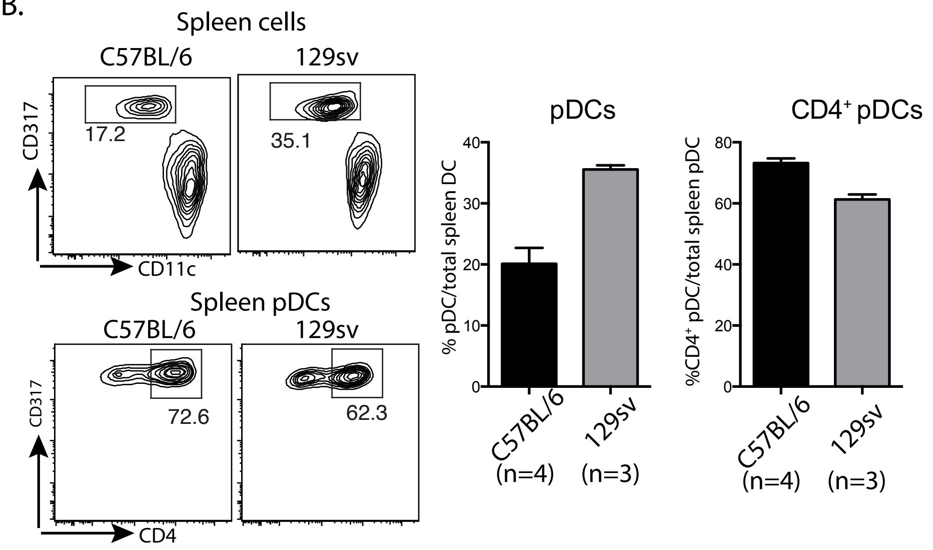

SF4B. 129sv mice have increased pDC compartment. Spleen cells were prepared from C57BL/6 mice and 129sv mice. Cells were stained for surface markers. FACS plot show pDCs in total gated DCs (upper panel) and $CD4^+$ pDCs in gated spleen pDCs (lower panel). Bar graphs show the percentage of pDCs and $CD4^+$ pDCs.
